# Application of a Two-Analyte Integrated Population Pharmacokinetic Model to Evaluate the Impact of Intrinsic and Extrinsic Factors on the Pharmacokinetics of Polatuzumab Vedotin in Patients with Non-Hodgkin Lymphoma

**DOI:** 10.1007/s11095-020-02933-6

**Published:** 2020-12-01

**Authors:** Dan Lu, Tong Lu, Rong Shi, Leonid Gibiansky, Priya Agarwal, Colby S. Shemesh, Randall C. Dere, Uzor Ogbu, Jamie Hirata, Pascal Chanu, Sandhya Girish, Jin Yan Jin, Chunze Li, Dale Miles

**Affiliations:** 1 Department of Clinical Pharmacology, Genentech, Inc, South San Francisco, California USA; 2 Genentech Research and Early Development, 1 DNA Way, MS46-3a, South San Francisco, California 94080 USA; 3 QuantPharm LLC, North Potomac, Maryland USA; 4 Department of Bioanalytical Sciences, Genentech, Inc, South San Francisco, California USA; 5 Product Development Oncology, Genentech, Inc, South San Francisco, California USA; 6 Department of Clinical Pharmacology, Genentech, Inc/F. Hoffmann-La Roche Ltd, Lyon, France

**Keywords:** antibody-drug conjugate, integrated two-analyte, non-Hodgkin lymphoma, population pharmacokinetics

## Abstract

**Purpose:**

The established two-analyte integrated population pharmacokinetic model was applied to assess the impact of intrinsic/extrinsic factors on the pharmacokinetics (PK) of polatuzumab vedotin (pola) in patients with non-Hodgkin lymphoma (NHL) following bodyweight-based dosing.

**Methods:**

Model simulations based on individual empirical Bayes estimates were used to evaluate the impact of intrinsic/extrinsic factors as patient subgroups on Cycle 6 exposures. Intrinsic factors included bodyweight, age, sex, hepatic and renal functions. Extrinsic factors included rituximab/obinutuzumab or bendamustine combination with pola and manufacturing process. The predicted impact on exposures along with the established exposure-response relationships were used to assess clinical relevance.

**Results:**

No clinically meaningful differences in Cycle 6 pola exposures were found for the following subgroups: bodyweight 100–146 kg *versus* 38–<100 kg, age ≥ 65 years *versus* <65 years, female *versus* male, mild hepatic impairment *versus* normal, mild-to-moderate renal impairment *versus* normal. Co-administration of rituximab/obinutuzumab or bendamustine, and change in the pola manufacturing process, also had no meaningful impact on PK.

**Conclusions:**

In patients with NHL, bodyweight-based dosing is adequate, and no further dose adjustment is recommended for the heavier subgroup (100–146 kg). In addition, no dose adjustments are recommended for other subgroups based on intrinsic/extrinsic factors evaluated.

**Electronic supplementary material:**

The online version of this article (10.1007/s11095-020-02933-6) contains supplementary material, which is available to authorized users.

## Introduction

Polatuzumab vedotin (pola) is a CD79b-directed antibody-drug conjugate (ADC) that targets proliferating B cells and contains the small-molecule, anti-mitotic agent, mono-methyl auristatin E (MMAE). MMAE has up to 100–1000 times more potency than vincristine (1,2). MMAE is covalently conjugated to anti-CD79b IgG1 monoclonal antibody at the site of reduced disulfide bonds in the hinge region via a protease-cleavable linker, maleimidocaproyl-valine-citrulline-p-aminobenzyloxycarbonyl (MC-vc-PAB) (2,3). Upon binding of the monoclonal antibody to CD79b, a signaling component of the B cell receptor (4), pola is internalized to the major histocompatibility complex class II positive compartment and the linker is cleaved by proteases for subsequent delivery of MMAE into the CD79b-positive lymphoma cells. MMAE binds to microtubules, inhibiting cell division and inducing apoptosis preferentially in proliferating tumor cells (5).

Pola was approved in 2019 by the United States Food and Drug Administration (FDA), indicated in combination with bendamustine and a rituximab (R) product (BR) for the treatment of adult patients with relapsed or refractory (R/R) diffuse large B cell lymphoma (DLBCL), not otherwise specified, after at least two prior therapies (6). Pola has also been approved by other regulatory agencies including the European Medicines Agency. The recommended dose of pola is 1.8 mg/kg as an intravenous infusion over 90 min every 21 days for six cycles; subsequent infusions after the first cycle may be administered over a shorter duration of 30 min if the previous infusion is tolerated (6).

The pharmacokinetics (PK) of ADCs are relatively complex because they contain both large and small-molecule components (7). Three key analytes were measured across studies: total antibody, conjugate (evaluated as antibody-conjugated MMAE [acMMAE]; i.e., MMAE that is conjugated to anti-CD79b antibody]), and unconjugated MMAE (8). A two-analyte (acMMAE and unconjugated MMAE) integrated population PK (popPK) model, based on data from 460 patients in four clinical studies using a liquid formulation of pola (manufacturing process v0.1), has been developed and validated (9). The model was able to describe the PK of acMMAE and unconjugated MMAE following repeated administration of pola 0.1–2.4 mg/kg every 3 weeks (Q3W) both as monotherapy and combination therapy in patients with non-Hodgkin lymphoma (NHL). Based on a stepwise covariate assessment, for acMMAE, the integrated popPK model indicated bodyweight, sex, race (Asian *versus* non-Asian), B cell count, tumor sum of product of dimensions, serum albumin level, R or obinutuzumab (G) combination (*versus* single-agent treatment), and treatment-naïve status (*versus* R/R) as statistically significant covariates. For unconjugated MMAE, bodyweight, sex, serum albumin level, R or G combination (*versus* single-agent treatment), treatment-naïve status (*versus* R/R), hepatic function status, and Eastern Cooperative Oncology Group (ECOG) score (ECOG = 0 *versus* ≥1) were statistically significant covariates.

In this paper, the established acMMAE-MMAE integrated popPK model (9) was further used to evaluate the magnitudes of impacts on the Cycle 6 exposures of acMMAE and unconjugated MMAE for subgroups of patients, based on the intrinsic and extrinsic factors (i.e., bodyweight, age, sex, hepatic or renal impairment, combinations [rituximab/obinutuzumab (R/G) or bendamustine], and pola manufacturing process). The predicted impact on exposures along with the established exposure-response relationships (10) were used to assess the clinical relevance and inform the United States Prescribing Information (USPI) label (6) for the dosing of pola in patient subgroups.

## Methods

### Study Design and Data

At the time of the original submission to United States FDA in 2018, PK data were available from seven pola clinical studies in patients with NHL, including phase I/Ib DCS4968g [NCT01290549]; phase II GO27834 [NCT01691898, ROMULUS]; phase Ib/II GO29365 [NCT02257567]; phase Ib/II GO29044 [NCT01992653]; phase Ib/II GO29833 [NCT02611323], phase Ib/II GO29834 [NCT02600897], and phase Ib/II BO29561 [NCT02729896]. As shown in Table I, these studies used pola material manufactured by two different processes: v0.1-derived drug product (DP) (liquid formulation) and v1.0-derived DP (lyophilized solid formulation used for commercialization). PK concentrations of acMMAE and unconjugated MMAE were quantified at pre-specified timepoints, with either intensive or less intensive PK sampling schedules (9). Among these studies, data from 460 patients enrolled in four studies (DCS4968g, GO27834, GO29365 [excluding Arm G] and GO29044) who received the v0.1-derived DP (Table I) were used to build the popPK model, as reported previously (9). Study GO29365 is the pivotal study supporting the approved indication (6). This study initially used the v0.1-derived DP to support approval. Later, after the manufacturing process was modified to yield the v1.0 DP for commercialization, study GO29365 was amended to add Arm G (*N* = 42 patients; pola in combinations with BR) with the major objectives to evaluate the PK, safety and efficacy of the v1.0 DP in the approved combination. In addition, the v1.0-derived DP was used in the following three ongoing or completed studies: GO29833 (*N* = 27), GO29834 (*N* = 47) and BO29561 (*N* = 34) (Table I). To evaluate the PK comparability of the v0.1 and v1.0-derived DP, the integrated popPK model developed based on v0.1-derived DP (9) was used to project pola exposure for patients receiving v1.0-derived DP as an external evaluation. This external evaluation included the following two datasets: 1) 42 patients in GO29365 Arm G and 2) 106 pooled patients in studies GO29833/GO29834/BO29561. As some studies, including GO29365, are ongoing, the data cut for the analysis reported here is March 2019.

**Table 1:** Studies Included in the Population Pharmacokinetic Analysis and the Version of Drug Product Received

Study number	Indication	Arms	Dose, mg/kg	Total number of patients	Drug product	PopPK analysis
DCS4968g	R/R B-NHL R/R CLL^a^	Pola only Pola + R	0.1 to 2.4	460	Liquid (v0.1) 100 mg/10 mL	Model development
GO27834 (ROMULUS)	R/R DLBCL R/R FL	Pola + R Pola + G	1.8, 2.4			
GO29365 (excluding Arm G)	R/R DLBCL R/R FL	Pola + BR Pola + BG	1.8			
GO29044	1st line DLBCL	Pola + R-CHP Pola + G-CHP	1.0, 1.4, 1.8, 2.4			
GO29833	R/R DLBCL R/R FL	Pola + G/R + Ven	1.4, 1.8	106	Lyophilized (v1.0) 170 mg/vial	External assessment for material bridging
GO29834	R/R DLBCL R/R FL	Pola + G/R + Lena	1.4, 1.8			
BO29561	R/R DLBCL R/R FL	Pola + G/R + Atezo	1.4, 1.8			
GO29365 (Arm G)	R/R DLBCL	Pola + BR	1.8	42	Lyophilized (v1.0) 140 mg/vial	

Note that data from studies GO29833, GO29834 and BO29561 were pooled for this analysis and reported in this document; data for Arm G of study GO29365 are reported separately

^a^CLL data were not included for population PK analysis for NHL patients reported in this paper

Abbreviations: Atezo, atezolizumab; BG, bendamustine and obinutuzumab; BR, bendamustine and rituximab; CHP, cyclophosphamide, doxorubicin, prednisone; CLL, chronic lymphocytic leukemia; DLBCL, diffuse large B cell lymphoma; FL, follicular lymphoma; G, obinutuzumab; Lena, lenalidomide; NHL, non-Hodgkin’s lymphoma; pola, polatuzumab vedotin; popPK, population pharmacokinetics; R, rituximab; R/R, relapsed or refractory; Ven, venetoclax

### Bioanalytical Methods

As previously reported (9), acMMAE concentrations in human plasma were measured using validated immunoaffinity capture and liquid chromatography with tandem mass spectrometric detection; unconjugated MMAE concentrations were measured using a validated protein precipitation/liquid chromatography method, with tandem mass spectrometry detection. The lower limits of quantitation (LLOQ) were 0.359 ng/mL (0.50 nM) MMAE for acMMAE, and 0.0359 ng/mL (0.05 nM) for unconjugated MMAE. In addition, total antibody concentrations in human serum were quantified using a validated enzyme-linked immunosorbent assay method, with a LLOQ of 50 ng/mL.

### Analysis of the Impact of Intrinsic and Extrinsic Factors as Patient Subgroups on PK Using the Established popPK Model

The two-analyte integrated popPK model (9) was applied to simulate the individual acMMAE and unconjugated MMAE exposures at Cycle 6, after the assumed pola dosing of 1.8 mg/kg Q3W. These simulations were based on the individual PK parameters adjusted by partial covariate correction (pCC) or complete covariate correction (cCC) (refer to “Detailed description of cCC and pCC methods” section). The analysis was conducted via nonlinear mixed-effects modelling with NONMEM software, v.7.4 (ICON Clinical Research LLC, Gaithersburg, MD, USA). Graphical and all other statistical analyses, including evaluation of NONMEM outputs, were performed using R v.3.5.3.

As shown in Table I, multiple studies were included for this analysis, with pola used either as a single agent or in combination with different agents, and administered to patients with different sub-types of NHL (e.g., follicular lymphoma, DLBCL) for different lines of therapy (e.g., previously untreated, R/R). To enable a robust and unbiased assessment of the subgroups of interest using data across studies/cohorts for the analysis, correction for possible imbalances in known covariates impacting pola PK was made using pCC or cCC approaches. The simulations were used to evaluate the impact of the intrinsic and extrinsic factors as patient subgroups on pola PK exposures, including the area under the concentration-time curve (AUC) and maximum concentration (C_max_) values for acMMAE and MMAE at Cycle 6. The intrinsic factors include bodyweight [heavier patients (100–146 kg) *versus* others (38–100 kg)], age [elderly patients (≥65 years) *versus* others (<65 years)], sex [males *versus* females], hepatic impairment status [mild hepatic impairment *versus* normal], and renal impairment status [mild-to-moderate renal impairment *versus* normal]. The extrinsic factors include drug combinations [with or without R/G or bendamustine] and pola manufacturing process [v0.1-derived DP *versus* v1.0-derived DP].

The impact on PK of the intrinsic and extrinsic factors mentioned above (with the exception of the manufacturing process) was assessed using data from the 460 patients in the popPK building dataset (DCS4968g, GO27834, GO29365 [excluding Arm G] and GO29044), who received v0.1-derived DP. Cycle 6 exposures were simulated based on the individual PK parameters that were derived by the population mean estimates (THETAs in the NONMEM code) and individual empirical Bayes estimates (EBE) of random effects from the established popPK model (9), and the partially corrected covariates (the pCC method; see “Detailed description of cCC and pCC methods”). The derived Cycle 6 exposures were compared for the subgroups of interest. As an additional supportive assessment, the results from the previously reported popPK sensitivity analysis (9) to assess the magnitude of impact of each statistically significant covariate on PK exposures (Cycle 6, AUC and C_max_ after 1.8 mg/kg pola Q3W) at the population level following 1.8 mg/kg Q3W were also summarized side by side in this paper. Specifically, for continuous covariates, the simulated exposure for patients with extreme values of certain covariate (2.5th and 97.5th percentiles) and reference values of all other covariates were compared with the exposure for the typical patient (i.e., subject with all covariates fixed at reference values or categories). Similarly for the categorical covariates, the simulated exposure in a patient with one category was compared with the exposure from the typical patient, with other covariates fixed at the reference values. The population predictions were used for the sensitivity analysis, that is, subjects with same covariate value had the same PK exposure, ignoring the variabilities between subjects.

To assess the impact of manufacturing process on PK, the data included both the popPK building dataset with v0.1-derived DP and the two external datasets with v1.0-derived DP: GO29365 Arm G (42 patients) and pooled GO29833/GO29834/BO29561 studies (106 patients). The following approaches were utilized for the assessment of manufacturing process. First, Goodness-of-Fit plots were generated to evaluate graphically whether the model built by PK data from v0.1-derived DP can describe the external datasets with v1.0-derived DP, by using the post-hoc Bayesian projection of the established popPK model (9). Second, the individual “completely adjusted” PK parameters were derived by the population mean estimates (THETAs), the individual EBE of random effects from post-hoc Bayesian projection, and the completely corrected covariates (the cCC method; see “Detailed description of cCC and pCC methods”); the Cycle 6 exposures were simulated for patients with v0.1-derived DP and v1.0-derived DP using the adjusted individual PK parameters, and were compared between the two DPs. Third, the PK time-profiles were simulated and compared between the two DPs: a population simulation was applied to simulate the PK profiles for patients taking v1.0-derived DP based on the population mean and variability estimates (THETAs and OMEGAs in the NONMEM code) from the established popPK model (9), the actual dosing log and sampling time, and the baseline covariate values for each individual. These population simulations were compared with observed PK data graphically by prediction corrected-visual predictive check (pc-VPC), and numerically by normalized prediction distribution error (NPDE) assessment (11–13) (see detailed methods descriptions of pc-VPC and NPDE in Supplementary methods). The same set of analyses were conducted for the two external datasets separately. No sensitivity analyses were performed for manufacturing process, as it was not assessed as a covariate in the popPK model, given that only the v0.1-derived DP process was used for model development.

For all the above analysis, the established exposure-response relationships (10) were used to justify whether the PK differences from the assessments above are clinically relevant requiring a dose adjustment for certain subgroups.

### Detailed Description of cCC and pCC Methods

As the cCC and pCC methods are popPK-based novel approaches that were firstly applied in the current analysis, and are fundamental to all analyses in this report; a detailed description is provided below.

The cCC method utilized the population mean estimates (THETAs), individual EBE of the random effects from the established popPK model (9), and the reference values of known PK covariates to derive the “completely adjusted” individual PK parameters to generate exposure simulations for comparison. To apply cCC, the factor of interest should have no correlation with the known PK covariates. This lack of correlation was confirmed for the extrinsic factor of manufacturing process. External datasets (i.e., not a part of the model building) were used for the assessment of the lyophilized material in comparison with the liquid material used in the popPK model building dataset. Therefore, the cCC approach is considered necessary to enable pooling across studies/cohorts for a robust and unbiased assessment of the impact of manufacturing process on pola PK, by eliminating any impacts on PK caused by potential imbalances of known PK covariates between the model building and external datasets.

The pCC method used the same approach as the cCC method, except that covariates were “partially” corrected (i.e., correction to reference values is only done for certain known PK covariates in the popPK model that are not correlated with the subgroup factor being assessed), whereas the other covariates were set to the individual values, to derive the “partially adjusted” individual PK parameters to generate exposure simulations for comparison. The pCC approach is also considered necessary for the unbiased analysis of pooled data across studies/cohorts to assess the impact of intrinsic and extrinsic factors on PK, by removing the impact on PK from the potentially unbalanced covariates. When assessing the impact of each of the following factors on pola PK: bodyweight, age, sex, hepatic function, renal function, bendamustine combination, all known PK covariates took the individual patients’ values except that R/R status (*versus* previously untreated) and R/G co-administration (*versus* pola single-agent) were set to reference values, that is, R/R status and co-administered with R/G. Correction by the pCC approach for R/R status and R/G co-administration status, which are covariates known to impact PK (9), enabled the pooling of data from different combination status (pola single agent and in co-administration with R/G) and disease status (previously untreated and R/R) in the analysis. Individual patient values for other covariates were used so that any underlying correlations were accounted for in the simulation. For example, hepatically impaired patients tend to have lower albumin levels (14); therefore, the use of individual values of albumin as a covariate in the simulation could more accurately reflect the overall impact of hepatic function on PK. When assessing the impact of R/G co-administration on pola PK, all patients were assumed to be R/R in the simulation, while individual patient values for all the other known PK covariates were used. Similarly, all patients were assumed to take pola in combination with R/G in the simulation, when assessing the impact of R/R *versus* previously untreated status on pola PK (reported separately in (18)).

Based on individual PK parameters derived from the pCC or cCC method (i.e., the “adjusted” individual PK parameters), the Cycle 6 AUC and C_max_ were simulated, assuming pola 1.8 mg/kg Q3W dosing for six cycles, and compared between the subgroups of interest by computing geometric mean ratios (GMR) and their 90% confidence interval (CI). It is worth noting that pre-specified bioequivalence criteria were not applied for any of these analyses, including the assessment of impact of material on pola PK, as the analysis was based on EBE of PK parameters from multiple studies that were not pre-designed with the intent of assessing bioequivalence among subgroups. Cycle 6 exposures were chosen to represent the exposure parameters after repeated Q3W dosing with a maximum of six cycles, which is theoretically the maximum exposure for this dosing regimen. For some factors that have more than two subgroups, for example, multiple subgroups of organ function impairment, the comparison was only made between each non-reference group relative to the reference group. The reference group typically has a large number of patients who received the label dose, and has more firmly established PK parameters. This approach supported the overall analysis objective, which was to determine if a label dose adjustment was needed for each of the non-reference subgroup.

PopPK parameter shrinkage is a potential issue for all the analyses that use the individual EBE of the random effects, including the pCC and cCC methods. Therefore, for material assessment using external datasets, a population simulation approach (VPC, NPDE), which is not subject to shrinkage as no individual EBE was involved, was performed as an additional analysis to support the cCC method. For other subgroups that were not related to material assessment and were evaluated using only the popPK model building dataset (*N* = 460), the validity of the pCC approach was justified by the low parameter shrinkage (<22%) from the popPK model for the key inter-individual random effect parameters driving the calculation of AUC and C_max_ for acMMAE and unconjugated MMAE (9).

## Results

### The Impact of Intrinsic Factors on Pola PK

#### Bodyweight

Given that pola dosing is based on bodyweight, there is a risk of overdosing patients with a higher bodyweight considering that the power coefficient is less than one (0.73) for the effect of bodyweight on acMMAE clearance (9). As reported previously (9), based on the sensitivity analyses of the impact of bodyweight after accounting for bodyweight-based dosing, bodyweight remained the most influential covariate on PK exposures of acMMAE and unconjugated MMAE (Cycle 6 AUC or C_max_). However, the magnitude of the impact of extreme values of bodyweight (2.5th and 97.5th percentile) on Cycle 6 C_max_ and AUC were < 25% for acMMAE and < 40% for unconjugated MMAE, of the corresponding values for a typical patient with the reference bodyweight. Given that a typical patient with a high bodyweight (e.g., upper 97.5th percentile) showed a trend for higher exposures by bodyweight-based dosing in the sensitivity analysis, which could potentially be associated with safety concerns, the exposures for the subgroup of heavy patients were compared with the rest of the patients using pCC-based simulation.

The pCC-based analysis showed that acMMAE exposures were slightly higher (8% for AUC; 17% for C_max_) among patients with bodyweight ≥100 kg (*N* = 59; range, 100–146 kg) than among patients with bodyweight <100 kg (*N* = 401; range, 38–<100 kg), and unconjugated MMAE exposures were moderately higher (27% for both AUC and C_max_; Table II). These magnitudes of difference were small compared with the inter-individual variability (IIV) as indicated by coefficient of variation (CV%) of the geometric mean (GM) of AUC and C_max_ for each analyte (13–22% for acMMAE and 38–50% for unconjugated MMAE; Table II). Also, the magnitudes of difference were not expected to have a clinically relevant impact on safety, based on the exposure–safety analysis ((10); see details in the Discussion section). Taken together, bodyweight-based dosing is justified and dose capping for relatively heavy patients included in this analysis (bodyweight of 100–146 kg) is not recommended based on the currently available data.

**Table II:** Comparison of Covariate-Corrected Exposures by Bodyweight and Age Categories, and Sex

Exposure	Statistics	Bodyweight		Patient age, years				Sex	
		<100 kg (*N* = 401)	≥100 kg (*N* = 59)	20–65 (*N* = 187)	65–74 (*N* = 187)	75–84 (*N* = 76)	85–89 (*N* = 10)	Females (*N* = 188)	Males (*N* = 272)
acMMAE parameters									
AUC, ng*day/mL	GM (CV%)	2880 (20)	3110 (22)	2950 (19)	2830 (23)	2950 (18)	3120 (18)	2990 (21)	2860 (20)
	GMR (90% CI)	1.08^a^ (1.03, 1.14)		ref	0.96 (0.93, 0.99)	1.00 (0.96, 1.04)	1.06 (0.95, 1.18)	0.96 (0.93, 0.99)^b^	
C_max_, ng/mL	GM (CV%)	720 (15)	842 (13)	758 (15)	726 (16)	704 (14)	693 (12)	770 (15)	711 (15)
	GMR (90% CI)	1.17 (1.14, 1.21)^a^		ref	0.96 (0.93, 0.98)	0.93 (0.90, 0.96)	0.91 (0.85, 0.98)	0.92 (0.90, 0.94)^b^	
Unconjugated MMAE parameters									
AUC, ng*day/mL	GM (CV%)	20.6 (50)	26 (39)	20.2 (47)	21.5 (50)	23.1 (51)	20.1 (56)	20.7 (45)	21.5 (52)
	GMR (90% CI)	1.27 (1.15, 1.39)^a^		ref	1.06 (0.98, 1.2)	1.14 (1.02, 1.28)	0.995 (0.72, 1.38)	1.04 (0.97, 1.12)^b^	
C_max_, ng/mL	GM (CV%)	1.91 (45)	2.43 (38)	1.91 (43)	2.01 (45)	2.03 (46)	1.71 (50)	1.94 (41)	1.98 (47)
	GMR (90% CI)	1.27 (1.16, 1.39)^a^		ref	1.05 (0.97, 1.13)	1.06 (0.96, 1.17)	0.89 (0.67, 1.2)	1.02 (0.95, 1.09)^b^	

Individual exposures were computed for all patients following polatuzumab vedotin 1.8 mg/kg Q3W dosing for 6 cycles using partial covariate correction. CV was computed as standard deviation of a log-transformed variable

^a^The reference arm for the GMR calculation is bodyweight <100 kg

^b^The reference arm for the GMR calculation is female

Abbreviations: acMMAE, antibody-conjugated monomethyl auristatin E; AUC, area under concentration-time curve; CI, confidence interval; C_max_, maximum concentration; CV, coefficient of variation; GM, geometric mean; GMR, geometric mean ratio; MMAE, monomethyl auristatin E; Q3W, every 3 weeks; ref., reference for GMR calculation for age category comparisons

#### Age

Pola is approved for patients with R/R DLBCL, which is more common in older patients (15). Age was not identified as a statistically significant covariate in the popPK model. PK results for patients aged <65 years (*N* = 187; range, 20–65 years) and ≥ 65 years (*N* = 273; which is further grouped for 65–74 years [*N* = 187], 75–84 years [*N* = 76], and 85–≤89 years [*N* = 10]) showed similar exposures for both acMMAE and unconjugated MMAE (<15% differences for AUC and C_max_ for each subgroup compared with age < 65 years as reference group; Table II), suggesting that dose adjustment based on age is not warranted based on the analysis across the age range of 20–89 years.

#### Male *Versus* Female

Sex is a statistically significant covariate for some PK parameters of acMMAE (non-specific time-dependent linear clearance [CL_INF_] and central volume of distribution [V_1_]) and unconjugated MMAE (relative fraction of formation [FRAC_NS_]) (9). Male patients have slightly lower exposures of acMMAE and unconjugated MMAE compared with female patients. Based on the sensitivity analysis, acMMAE and unconjugated MMAE are less than 17% lower for male patients compared with female patients for AUC and C_max_. Furthermore, pCC-based simulation of Cycle 6 exposures (AUC, C_max_ at 1.8 mg/kg Q3W) suggested that PK exposures for males (*N* = 272) were similar (<10% differences for AUC and C_max_) for acMMAE and unconjugated MMAE compared with females (*N* = 188), after adjusting for impact of bodyweight (Table II). The magnitudes of these impacts are small, and well within the IIV as indicated by CV% of GM of AUC and C_max_ for each analyte of 15–21% for acMMAE and 41–52% for unconjugated MMAE (Table II). Therefore, dose adjustment based on sex is not warranted.

#### Hepatic Function Impairment

Based on the popPK covariate analysis, among the baseline laboratory chemistry markers of liver function such as aspartate aminotransferase (AST), alanine aminotransferase (ALT), total bilirubin, and albumin level, only albumin level was identified as a statistically significant covariate of acMMAE CL_INF_ and unconjugated MMAE FRAC_NS_ (9). Based on the sensitivity analysis (9), the magnitude of albumin effect was <8% for acMMAE exposures and < 23% for unconjugated MMAE exposures comparing hypothetical patients with extreme albumin levels (at 2.5th and 97.5th percentiles) with a typical patient, with lower acMMAE and higher unconjugated MMAE exposures for patients with lower albumin level.

In addition to these individual laboratory chemistry markers related to hepatic function, the National Cancer Institute (NCI) criteria (which considers bilirubin and AST levels) (16) was assessed as a categorical covariate in the popPK model (Supplementary Table I). Based on the protocol exclusion criteria, patients with AST or ALT >2.5 upper limit of normal (ULN) or total bilirubin ≥1.5 ULN were excluded. Patients with documented Gilbert’s disease were considered if total bilirubin was ≤3 ULN. Overall, 399 patients had normal hepatic function, 54 patients had mild hepatic impairment, and two patients had moderate impairment. An additional three patients were classified as having moderate hepatic impairment based on elevated total bilirubin, which was actually due to Gilbert’s disease but not hepatic impairment.

In the popPK model, hepatic function, based on NCI criteria (normal *versus* mild or moderate hepatic impairment), was not a statistically significant covariate of acMMAE PK parameters, but was a statistically significant covariate on FRAC_NS_ of unconjugated MMAE. Patients with mild or moderate hepatic impairment demonstrated 19% higher FRAC_NS_ for unconjugated MMAE (9). Sensitivity analyses suggested that unconjugated MMAE exposures were slightly higher (25.5% for AUC, 22.3% for C_max_) in patients with hepatic function impairment (mild or moderate) than in typical patients with normal hepatic function (9).

By pCC-based simulation, exposures in 54 patients with mild hepatic impairment (AST >1–2.5 upper limit of normal [ULN] or total bilirubin of >1–1.5 ULN) *versus* 399 patients with normal hepatic impairment suggested that acMMAE exposures are similar (<5% difference), while unconjugated MMAE exposures were higher in patients with mild hepatic impairment (40% for AUC, 34% for C_max;_ Table III). These magnitudes of differences were less with the IIV of unconjugated MMAE as indicated by CV% of GM (43–49% for C_max_ and 47–57% for AUC; Table III). Given that the individual albumin level was used for the pCC simulation of hepatic impairment, the higher unconjugated MMAE exposures in patients with mild hepatic impairment may be an outcome from both the hepatic impairment (by NCI criteria) and the decreased albumin level, which are correlated with each other. These magnitudes of difference were not expected to have a clinically relevant impact on safety, based on the exposure–safety analysis ((10); see details in the Discussion section).

**Table III:** Comparison of Covariate-Corrected Exposures by Hepatic and Renal Function

Exposure	Statistics	Hepatic function		Renal function		
		Normal (*N* = 399)	Mild impairment (*N* = 54)	Normal (*N* = 185)	Mild impairment (*N* = 161)	Moderate impairment (*N* = 109)
acMMAE parameters						
AUC, ng*day/mL	GM (CV%)	2920 (20)	2810 (24)	2930 (19)	2910 (21)	2890 (22)
	GMR (90% CI)	0.96 (0.91, 1.02)^a^		ref	0.99 (0.96, 1.03)	0.99 (0.94, 1.03)
C_max_, ng/mL	GM (CV%)	738 (15)	710 (17)	763 (15)	736 (15)	694 (13)
	GMR (90% CI)	0.96 (0.92, 1.0)^a^		ref	0.96 (0.94, 0.99)	0.91 (0.88, 0.94)
Unconjugated MMAE parameters						
AUC, ng*day/mL	GM (CV%)	20.4 (47)	28.5 (57)	20.4 (43)	22 (54)	21.7 (52)
	GMR (90% CI)	1.40 (1.22, 1.6)^a^		ref	1.08 (0.99, 1.18)	1.06 (0.97, 1.17)
C_max_, ng/mL	GM (CV%)	1.9 (43)	2.55 (49)	1.95 (39)	2.01 (50)	1.97 (45)
	GMR (90% CI)	1.34 (1.2, 1.51)^a^		ref	1.03 (0.95, 1.12)	1.01 (0.93, 1.10)

Individual exposures were computed for patients with normal hepatic function and mild hepatic impairment, and for normal renal function (CrCL >90 mL/min), mild (CrCL 60–90 mL/min) or moderate renal impairment (CrCL 30–60 mL/min) following polatuzumab vedotin 1.8 mg/kg Q3W dosing for 6 cycles using partial covariate correction. CV was computed as standard deviation of a log-transformed variable

^a^The reference arm for the GMR calculation is normal hepatic function

Abbreviations: acMMAE, antibody-conjugated monomethyl auristatin E; AUC, area under concentration-time curve; CI, confidence interval; C_max_, maximum concentration; CrCL, creatinine clearance; CV, coefficient of variation; GM, geometric mean; GMR, geometric mean ratio; MMAE, monomethyl auristatin E; Q3W, every 3 weeks; ref., reference for the GMR calculation for PK comparison in groups of renal functions

Thus, starting dose adjustment is not warranted for patients with mild hepatic impairment. No PK- or safety-related conclusions can be drawn for patients with moderate or severe hepatic impairment (total bilirubin >1.5–3 ULN and > 3 ULN, respectively, where enzyme elevation is not due to Gilbert’s syndrome or underlying disease) due to limited data (*N* = 2 and 0, respectively). This was indicated in the current USPI as an unknown effect on the PK of acMMAE or unconjugated MMAE; it is recommended that treatment with pola should be avoided in these patient groups (6).

#### Renal Function Impairment

The estimated serum creatinine clearance (CrCL) based on the Cockcroft-Gault formula is an indicator of renal function. Based on the popPK analysis, CrCL was not identified as a statistically significant covariate of acMMAE or unconjugated MMAE PK parameters. When patients were grouped by renal function based on CrCL (17), renal function as a categorical covariate was also not identified as a statistically significant covariate in the popPK model (Supplementary Table II). Simulated exposures by the pCC procedure suggested that patients with mild (CrCL 60–89 mL/min; *N* = 161) or moderate renal impairment (CrCL 30–59 mL/min; *N* = 109) had similar acMMAE and unconjugated MMAE exposures (i.e., <10% differences in AUC and C_max_) to normal patients (CrCL ≥90 mL/min; *N* = 185; Table III). Based on the similar PK and safety profiles for these subgroups (Genentech, Inc. data on file), starting dose adjustment is not warranted in patients with mild or moderate renal impairment.

No PK and safety conclusions can be drawn for patients with severe renal impairment (CrCL 15–29 mL/min) or end-stage renal disease (ESRD) due to limited data (*N* = 3 and 0, respectively). This was indicated in the current USPI as an unknown effect on the PK of acMMAE or unconjugated MMAE.

### The Impact of Extrinsic Factors on Pola PK

#### Single-Agent Pola *Versus* Pola-R/G Combinations

Based on popPK covariate analysis, the R/G combination with pola (*versus* single-agent pola) was a statistically significant covariate on CL_INF_ and k_des_ (rate constant of decay of the linear time-dependent exponentially declining clearance with time) for acMMAE, and on FRAC_NS_ for unconjugated MMAE (9). Based on sensitivity analyses (9), pola-R/G combination was associated with slightly higher acMMAE exposures compared with pola monotherapy (18.4% higher for AUC; 1.3% higher for C_max_) and moderately lower unconjugated MMAE exposures (approximately 38% lower for both AUC and C_max_).

Consistent with the sensitivity analyses, simulated Cycle 6 exposures using the EBE of PK parameters by the pCC procedure (i.e., individual values for all covariates, except disease state, which was corrected to a reference of R/R disease), showed PK exposures in patients treated with pola + R (*N* = 229) *versus* single-agent pola (*N* = 68) to be moderately higher for acMMAE exposures (24% for AUC and 5% for C_max_) and moderately lower unconjugated MMAE exposures (37% for AUC and 40% for C_max_; Table IV). The magnitudes of these differences were less than the IIV as indicated by CV% of GM of AUC and C_max_ for each analyte (15–34% for acMMAE; 43–59% for unconjugated MMAE; Table IV). Based on the exposure-safety analysis ( (10); see details in the discussion section), these magnitudes of difference were not expected to have a clinically relevant impact on safety or efficacy. Similar magnitudes of impact for acMMAE and unconjugated MMAE were observed with G as those that were observed with R (Genentech, Inc. data on file). Thus, the risk of a clinically meaningful impact of R/G combination on pola PK is low, and the exposure differences were not considered clinically meaningful to justify any dose adjustment.

**Table IV:** Comparison of Covariate-Corrected Cycle 6 Exposures of acMMAE and Unconjugated MMAE According to Rituximab Administration and Bendamustine Administration

Exposure	Statistics	Rituximab administration^a^		Bendamustine administration^b^	
		No	Yes	No	Yes
		(N = 68)	(N = 229)	(N = 321)	(*N* = 139)
acMMAE parameters					
AUC, ng*day/mL	GM (CV%)	2350 (34)	2910 (18)	2870 (22)	3010 (16)
	GMR (90% CI)	1.24 (1.15, 1.33)^c^		1.05 (1.02, 1.08)^c^	
C_max_, ng/mL	GM (CV%)	696 (17)	729 (15)	737 (16)	728 (14)
	GMR (90% CI)	1.05 (1.01, 1.09)^c^		0.987 (0.964, 1.01)^c^	
Unconjugated MMAE parameters					
AUC, ng*day/mL	GM (CV%)	33.2 (58)	20.8 (49)	22.2 (52)	19.1 (42)
	GMR (90% CI)	0.627 (0.551, 0.713) ^c^		0.862 (0.799, 0.929) ^c^	
C_max_, ng/mL	GM (CV%)	3.22 (59)	1.92 (43)	2.05 (47)	1.77 (36)
	GMR (90% CI)	0.597 (0.525, 0.679) ^c^		0.864 (0.808, 0.923) ^c^	

^a^Individual exposures were computed for patients with and without co-administration of rituximab following polatuzumab vedotin 1.8 mg/kg Q3W dosing for six cycles using partial covariate correction. CV% was computed as standard deviation of a log-transformed variable. All patients were assumed to be relapsed or refractory

^b^Cycle 6 exposures were computed assuming polatuzumab vedotin 1.8 mg/kg Q3W for six cycles simulated from empirical Bayes estimates of individual PK parameters by partial covariate correction that partially corrected for two covariates (i.e., assumed all patients received R/G combination and had R/R status)

^c^The reference arm for GMR calculation is without rituximab or without bendamustine treatment

Abbreviations: acMMAE, antibody-conjugated monomethyl auristatin E; AUC, area under concentration-time curve; C_max_, maximum concentration; CV, coefficient of variation; GM, geometric mean; GMR, geometric mean ratio; MMAE, monomethyl auristatin E; PK, pharmacokinetic; Q3W, every 3 weeks; R/G, rituximab/obinutuzumab

#### Pola-Bendamustine Combination

Bendamustine in combination with pola was not identified as a statistically significant covariate on acMMAE or unconjugated MMAE PK parameters (9). PK results in patients who received pola with (*N* = 139) *versus* without bendamustine (*N* = 321) based on the simulated Cycle 6 exposures with pola 1.8 mg/kg Q3W using EBE of PK parameters by the pCC procedure showed similar exposures for acMMAE and unconjugated MMAE (<15% differences for AUC and C_max_; Table IV). Thus, the risk of bendamustine combination having a clinically meaningful impact on pola PK is low.

#### Manufacturing Process

The popPK model built based on liquid formulation well described the external PK data from Arm G of GO29365 and studies GO29833, BO29561 and GO29834 dosed with lyophilized material. The Goodness-of-Fit plots (Supplementary Figs. I and II) showed no obvious deficiencies, suggesting that the model described the observed data from the two external datasets well in general. Based on the “completely adjusted” individual PK parameters from cCC method, the simulated Cycle 6 exposures (AUC and C_max_) assuming pola 1.8 mg/kg Q3W dosing also showed similarity (<10% difference for acMMAE; <12% difference for unconjugated MMAE) for the v1.0-derived lyophilized DP and v0.1-derived liquid DP, with the confidence intervals for GMR falling within the 0.8–1.25 range, indicating material comparability (Table V). Based on the pc-VPC and NPDE from the population simulations, the integrated popPK model built on data from v0.1-derived DP (9) well projected the exposures for patients receiving the v1.0-derived DP. By pc-VPC assessment stratified by dosing schedule of Q3W or every 4 weeks, the median profile of the observed PK data for acMMAE and unconjugated MMAE from the 106 patients in studies GO29833, BO29561 and GO29834 (solid red line) was in alignment with the predicted median values and 80% CI of the median values (red shaded area) from the popPK model (Supplementary Figures IIIa, IIIb and IV). Similar observations were found for 42 patients from Arm G of the GO29365 study (Supplementary Figures IIIc, IIId). As assessed numerically by the NPDE, there was an overall consistency between the observed PK observations and the model predicted values across different time points for acMMAE and unconjugated MMAE, for both the data from pooled studies BO29561, GO29833 and GO29834 and the data from Arm G of the Study GO29365 (Supplementary Table III). Taken together, there were no clinically meaningful differences in the PK of acMMAE and unconjugated MMAE after repeated dosing between the v1.0-derived lyophilized DP and v0.1-derived liquid DP.

**Table V:** Comparison of Covariate-Corrected Cycle 6 Exposures of acMMAE and Unconjugated MMAE According to the Polatuzumab Vedotin Drug Product Received

Exposure	Statistics	Drug product		
		v0.1-derived liquid (*N* = 460)	v1.0-derived lyophilized	
			GO29833, GO29834 and BO29561 studies (*N* = 106)	Arm G of the GO29365 study (*N* = 42)
acMMAE parameters				
AUC, ng*day/mL	GM (CV%)	3140 (18)	2870 (14)	2900 (15)
	GMR (90% CI)	ref	0.91 (0.89, 0.94)	0.92 (0.89, 0.96)
C_max_, ng/mL	GM (CV%)	801 (11)	746 (12)	742 (12)
	GMR (90% CI)	ref	0.93 (0.91, 0.95)	0.93 (0.90, 0.96)
Unconjugated MMAE parameters				
AUC, ng*day/mL	GM (CV%)	24.1 (43)	21.4 (44)	23 (47)
	GMR (90% CI)	ref	0.89 (0.82, 0.96)	0.95 (0.84, 1.08)
C_max_, ng/mL	GM (CV%)	2.21 (38)	1.99 (39)	2.12 (41)
	GMR (90% CI)	ref	0.90 (0.84, 0.97)	0.96 (0.86, 1.07)

Individual exposures were computed for all patients following polatuzumab vedotin 1.8 mg/kg Q3W dosing for six cycles using complete covariate correction based on covariate-corrected empirical Bayes estimates of individual pharmacokinetic parameters. CV was computed as standard deviation of a log-transformed variable

Abbreviations: acMMAE, antibody-conjugated monomethyl auristatin E; AUC, area under concentration-time curve; C_max_, maximum concentration; CV, coefficient of variation; DP, drug product; GM, geometric mean; GMR, geometric mean ratio; MMAE, monomethyl auristatin E; Q3W, every 3 weeks; ref., reference for the GMR calculation

## Discussion

Development of an integrated popPK model to simultaneously fit the PK of acMMAE and unconjugated MMAE after pola dosing in NHL patients (9) enabled the investigation of subgroups of clinical interest based on various intrinsic (bodyweight, age, sex, hepatic and renal impairment) and extrinsic factors (combination with R/G or bendamustine, manufacturing process) that could potentially impact acMMAE and MMAE PK. The acMMAE exposures are highly correlated with pola dose and acMMAE is considered as the key analyte driving efficacy and safety of pola to inform the dose justification. Unconjugated MMAE is a catabolite of the conjugate with much lower exposures (AUC for unconjugated MMAE is less than 3% of the AUC for acMMAE) (9). Unconjugated MMAE, when deconjugated from the antibody, lacks the B cell targeting conferred by conjugation to the anti-CD79b antibody, and is unlikely to be delivered to cancerous B-cells at a high enough quantity to meaningfully contribute to the cell killing. Thus, unconjugated MMAE is not considered as a major driver of efficacy but might be associated with safety risk given its high potency compared to traditional chemotherapy. The predicted impact of these intrinsic/extrinsic factors on post-hoc Cycle 6 exposures (AUC, C_max_) using pCC or cCC based simulations, together with the sensitivity analyses of the impact of significant covariates at extreme covariate values (2.5th and 97.5th percentiles), were used in conjunction with the established exposure-response relationships to assess whether dose adjustment is needed for any subgroups of patients based on these intrinsic/extrinsic factors. The impact of treatment-naïve disease status *versus* R/R status on pola PK was reported separately (18). The impact of CYP3A inhibitor/inducer and P-gp inhibitor/inducer on MMAE PK was assessed by a physiological-based PK approach (19) (Genentech, Inc. data on file) to inform the label, and were not included in this report.

### Dosing in the Subgroup of Heavier Patients

Given that the pola dose is based on bodyweight, there is a risk of overdosing for higher bodyweight patients considering that the power coefficient for the effect of bodyweight on acMMAE clearance is less than one (0.73). The impact of bodyweight was assessed mainly by comparing the Cycle 6 exposures, in which the correlation between bodyweight and gender was properly considered by using the individual covariate values for simulation, with all patients assumed to have R/R disease and who received pola + R/G combination (the pCC approach). Thus, this represented a potentially more realistic scenario to assess the magnitude of impact of bodyweight on pola PK and to guide the dosing in the subgroup of heavier patients (bodyweight ≥100 kg). The pCC approach is essential to allow unbiased analysis of pooled data across studies/cohorts, including different combination status (pola single agent and in combination with R/G) and disease status (previously untreated and R/R). The clinical relevance for higher exposures in this subgroup has been assessed in the context of exposure-response relationships (10). The exposure-response analysis indicated that the probability of grade ≥ 3 anaemia increased with increasing unconjugated MMAE exposure (*p* = 0.01 for AUC and *p* = 0.015 for C_max_), and the probability of grade ≥ 2 peripheral neuropathy increased with increasing acMMAE exposure (*p* = 0.003 for AUC and *p* = 0.011 for C_max_) (10). However, a 27% increase in unconjugated MMAE AUC (20.6–26.0 ng*day/mL) and C_max_ (1.9–2.4 ng/mL) in the heavier subgroup is not expected to result in a clinically meaningful increase in the incidence of grade ≥ 3 anaemia (3.8–4.5% and 3.8–4.7% on average, respectively), and 8% increase of acMMAE AUC (2880–3110 ng*day/mL) and 17% increase for C_max_ (720–842 ng/mL) is not expected to result in a clinically meaningful increase in the incidence of grade ≥ 2 peripheral neuropathy (17.8–20.3% and 17.9–23.7% on average, respectively). Thus, the current analysis justified bodyweight-based dosing, and dose capping for the relatively heavy patients included in the current analysis (bodyweight of 100–146 kg) is not warranted. However, caution should be taken in patients weighing >146 kg, given the limited clinical data currently available. Further data from the ongoing clinical trials of pola and from real-world studies will inform the dosing strategy in patients with extremely high bodyweight.

### Dosing in Patients with Hepatic or Renal Impairment

MMAE released from pola is expected to be predominantly excreted via a hepatobiliary pathway, as supported by the preclinical data in rats, showing that after intravenous dosing with tritium-radiolabeled pola in the MMAE portion, >95% of the radiolabeled dose was excreted in the faeces over 14 days (6). Thus, the risk of alteration in MMAE exposures is higher with hepatic impairment than renal impairment. However, given that MMAE is not extensively metabolized in liver and is mainly excreted unchanged in rat bile after pola dosing (6), and the unconjugated MMAE appears to follow formation-rate limited kinetics after pola dosing (9), magnitude of the exposure alteration for unconjugated MMAE may be low compared with compounds that are more extensively metabolized in the liver, or the compounds that follow elimination-rate limited kinetics. As discussed in the method section, the pCC approach was used for this analysis with all patients assumed to have R/R disease and received pola + R/G combination (the reference covariate values), which allowed unbiased analysis of pooled data across studies/cohorts. The individual values were used for other covariate variables to account for the potential underlying correlations between the subgroup of interests (hepatic or renal impairment) and the covariates (such as albumin) for a realistic simulation. The results suggest that mild impairment of hepatic function, or mild/moderate impairment of renal function, did not cause a clinically relevant increase on the unconjugated MMAE exposures after pola administration. For patients with mild hepatic impairment, the unconjugated MMAE exposures increased by 40% (20.4–28.5 ng*day/mL) for AUC and 34% for C_max_ (1.9–2.6 ng/mL) compared with patients with normal hepatic function, resulting in the increase of the probability of grade ≥ 3 anaemia from 3.8–4.9% on average, which is a relatively small change and is not expected be clinically meaningful. Clinical safety data also suggested no substantial decrease in tolerability (as indicated by dose intensity, dose reductions or dose delays) in patients with mild hepatic impairment (6,20). Although lower albumin (a covariate that is related to hepatic function, but is also affected by other factors related to patient overall health status) was associated with faster conjugate clearance, hepatic impairment based on NCI criteria is not identified as a statistically significant covariate on acMMAE PK parameters in the model. While a dose reduction to <1.8 mg/kg in mild hepatically impaired patients could reduce the unconjugated MMAE concentrations to the level close to the normal patients, the acMMAE levels, which are not significantly impacted by hepatic impairment (GMR = 0.96; Table III), would be reduced. Therefore, a dose reduction could potentially compromise efficacy in these patients based on a positive correlation between acMMAE exposure and efficacy based on exposure-response analyses, and thus is not recommended (10).

The strategy to assess PK of pola in patients with organ impairment enrolled into clinical safety/efficacy studies, without performing dedicated organ impairment studies, was informed by the published results for other FDA-approved ADCs and an internal evaluation of the real-world data (RWD) in DLBCL patients, as discussed below.

We first surveyed the strategies to inform the PK and label dose in patients with hepatic or renal impairment for a few FDA-approved ADCs, including brentuximab vedotin (BV) (21,22), trastuzumab emtansine (23,24), gemtuzumab ozogamicin (25) and inotuzumab ozogamicin (26,27). Similar to pola, the payloads of all of these ADCs were excreted mainly through biliary-faecal elimination. To assess the PK, either popPK approaches or dedicated studies were used, as summarized in Table VI. Among these ADCs, BV is quite similar to pola in its chemical composition (identical linker and cytotoxic MMAE payload) and *in vivo* disposition. Both drugs contain an IgG1 subclass of antibody portion, with identical linker, payload and interchain disulphide conjugation sites. They have demonstrated similar *in vitro* stability after incubation in human plasma, and rat studies have shown that MMAE released from both ADCs is mainly excreted via the biliary-faecal pathway (28). Both drugs were approved at the dose of 1.8 mg/kg Q3W with comparable unconjugated MMAE PK (6,22). Dedicated studies for BV in patients with hepatic or renal impairment have been conducted. It was found that conjugate exposures were slightly decreased in patients with hepatic impairment, potentially due to hypoalbuminemia and consequently faster antibody clearance; unconjugated MMAE exposures were apparently increased in patients with hepatic impairment or severe renal impairment, potentially due to increased conjugate clearance or decreased MMAE elimination, or a combination of both (21). Given the similar chemical composition and *in vivo* disposition, it would be reasonable to expect similar findings if dedicated hepatic or renal impairment studies were conducted for pola.

**Table VI:** Summary of Assessment Strategy for Five Approved Antibody–Drug Conjugates by Food and Drug Administration (FDA) Strategy and the United States Prescribing Information (USPI) Outcome (6,20–24,26)

	Brentuximab vedotin	Trastuzumab emtansine	Gemtuzumab ozogamicin	Inotuzumab ozogamicin	Polatuzumab vedotin
Strategy					
Hepatic	Dedicated study: Child-Pugh	Dedicated study: Child-Pugh	PopPK (conjugate): NCI criteria	PopPK (conjugate): NCI criteria	PopPK (acMMAE & MMAE): NCI criteria & exposure-response
Renal	Dedicated study: CrCL based criteria	PopPK (conjugate): CrCL based criteria	PopPK (conjugate): CrCL based criteria	PopPK (conjugate): CrCL based criteria	PopPK (acMMAE & MMAE): CrCL based criteria & exposure-response
Label: hepatic function					
Mild	Reduce dose from 1.8 to 1.2 mg/kg	No adjustment	No adjustment	No adjustment	No adjustment (1.8 mg/kg)
Moderate	Avoid	No adjustment	Unknown PK; higher risk of VOD	Unknown PK	Unknown PK; avoid
Severe	Avoid	Unknown	Unknown PK; higher risk of VOD	Unknown PK; avoid	Unknown PK; avoid
Label: renal function					
Mild	No adjustment	No adjustment	No adjustment	No adjustment	No adjustment
Moderate	No adjustment	No adjustment	No adjustment	No adjustment	No adjustment
Severe	Avoid	Unknown	Unknown	No adjustment	Unknown

Abbreviations: acMMAE, antibody-conjugated monomethyl auristatin E; CrCL, creatinine clearance; MMAE, monomethyl auristatin E; NCI, National Cancer Institute; popPK, population pharmacokinetics; VOD, veno-occlusive liver disease

To further assess the feasibility of conducting these dedicated organ impairment studies in DLBCL patients if needed, an exploratory analysis of RWD in the selected cohorts from the patient population with previously untreated DLBCL from the Flatiron^®^ database was performed (data cut March 2018). When the NCI criteria (16) of hepatic impairment was applied to a cohort of 1341 previously untreated DLBCL patients, it was found that 5.8% of patients had moderate or severe hepatic impairment and 8.8% of patients would have been excluded from the pola clinical studies if the inclusion/exclusion criteria related to AST/ALT and total bilirubin levels were applied (Table VII). Among the 118 patients that would be excluded, 28 patients had moderate impairment and 50 had severe hepatic impairment, and the remaining 40 patients (AST >2.5 x ULN; total bilirubin ≤ULN) belonged to the group with mild impairment. When the renal function impairment criteria based on CrCL (17) were applied to a cohort of 1269 previously untreated DLBCL patients, it was found that 5.1% of patients had severe renal impairment or ESRD, and 14.3% of patients would have been excluded from the pola clinical studies if the inclusion/exclusion criteria related to CrCL were applied (Table VIII). Among the 182 patients that would be excluded, 65 patients had severe renal impairment and 117 patients (CrCL of 30–40 mL/min) belonged to the group with moderate renal impairment. Based on the relatively low percentage inferred from RWD, we consider enrolling DLBCL patients with moderate or severe hepatic impairment (<6%) or with severe renal impairment (<6%) would be challenging, if dedicated studies were conducted.

**Table VII:** Proportions of Patients with Hepatic Impairment when NCI criteria^a^ and Pola Study Inclusion/Exclusion Criteria^b^ are Applied to a Real-World Cohort of 1341 Patients With Previously Untreated DLBCL from the Flatiron^®^ Database

Hepatic function	Total *N* = 1341
NCI criteria^a^ (group number)	
Normal (A): AST ≤ ULN; total bilirubin ≤ULN	1052 (78.4)
Mild impairment (B1): total bilirubin 1–1.5 x ULN	157 (11.7)
Mild impairment (B2): AST ≥ ULN, total bilirubin ≤ULN	49 (3.7)
Moderate impairment (C): total bilirubin 1.5–3 x ULN	28 (2.1)
Severe impairment (D): total bilirubin >3 x ULN	50 (3.7)
Liver Transplant (E)	0 (0)
Indetermined^c^	5 (0.4)
Pola study inclusion/exclusion criteria^b^	
Excluded: AST or ALT ≥2.5 ULN or total bilirubin ≥1.5 ULN	118 (8.8)
Included: AST and ALT <2.5 ULN and total bilirubin <1.5 ULN	1210 (90.2)
Indetermined^d^	13 (1.0)

Values shown are n (%)

^a^See Supplementary Table I for NCI criteria for hepatic function impairment

^b^Polatuzumab vedotin clinical trial exclusion criteria related to hepatic functions: AST or ALT ≥2.5 ULN or total bilirubin ≥1.5 ULN

^c^5 patients (0.4%) were excluded due to missing AST or total bilirubin data at baseline

^d^13 patients (1%) were excluded due to missing AST or ALT or total bilirubin data at baseline

Abbreviations: ALT, alanine aminotransferase; AST, aspartate aminotransferase; NCI, National Cancer Institute; ULN, upper limit of normal

**Table VIII:** Proportions of Patients with Renal Impairment when the Renal Function Criteria Based on Creatinine Clearance (CrCL)^a^ and Polatuzumab Vedotin (pola) Study Inclusion/Exclusion Criteria are Applied to a Real-World Cohort of 1269 Patients with Previously Untreated Diffuse Large B Cell Lymphoma from the Flatiron^®^ Database

Renal function	Total *N* = 1269
Based on creatinine clearance (CrCL)^a^	
Normal: CrCL ≥90 mL/min	406 (32.0)
Mild impairment: CrCL 60–89 mL/min	379 (29.9)
Moderate impairment: CrCL 30–59 mL/min	419 (33.0)
Severe impairment: CrCL 15–29 mL/min	64 (5.0)
ESRD: CrCL <15 mL/min	1 (0.1)
Based on pola study inclusion/exclusion criteria^b^	
Excluded: CrCL <40 mL/min	182 (14.3)
Included: CrCL ≥40 mL/min	1087 (85.7)

Values shown are n (%)

^a^From: Guidance for Industry. Pharmacokinetics in Patients with Impaired Renal Function – Study Design, Data Analysis, and Impact on Dosing and Labeling. U.S. Food and Drug Administration, 2010 (16)

^b^Pola study inclusion criteria were to enrol patients with CrCL ≥40 mL/min unless due to the underlying disease of lymphoma

Abbreviations: CrCL, creatinine clearance, ESRD, end-stage renal disease; pola, polatuzumab vedotin

The currently approved indication for BV is Hodgkin lymphoma and for pola is R/R DLBCL, which may imply a different risk/benefit profile when assessing the dose recommendation in patients with organ impairment based on PK in these subgroups. In the current FDA label, BV appears to have a relatively stringent dosing recommendations for patients with organ impairment (22) i.e., avoid treatment for patients with moderate and severe hepatic impairment and severe renal impairment, and reduce the dose from 1.8 mg/kg to 1.2 mg/kg for patients with mild hepatic impairment (Table VI). For pola in the current FDA label (6), it is recommended to avoid dosing for patients with moderate-to-severe hepatic impairment, but starting dose reduction is not recommended for patients with mild hepatic impairment; there is no dose recommendation for patients with severe renal impairment given the currently limited clinical data. Not recommending dose reduction for patients with mild hepatic impairment for pola is mainly due to risk/benefit considerations, as dosing reduction may compromise efficacy in R/R DLBCL, a disease with potentially poorer prognosis compared with Hodgkin lymphoma (the target indication for BV).

Taken together, a popPK-based strategy based on the data from patients enrolled in pola clinical studies were applied to assess PK in patients with hepatic or renal impairment, instead of a dedicated study. Due to limited data, no conclusions on PK could be drawn for the category of moderate and severe hepatic impairment, or severe renal impairment after pola treatment. The FDA label recommendation is to avoid the treatment in patients with moderate and severe hepatic impairment (6). No guidance is provided for renal impairment and the impact of severe renal impairment on pola PK is acknowledged as unknown. After the launch of pola, analyses of RWD may provide cumulative evidence of some safety outcomes in the pola-treated patients with moderate-to-severe hepatic impairment and severe renal impairment post-approval, which may inform dose recommendations for pola in these patient groups as data are accumulated.

### The Risk of Drug Interaction for the Pola + R/G Combination

In this study, we also assessed the impact of the pola + R/G combination as an extrinsic factor on pola PK. R and G binds specifically to CD20 expressed on >90% of B cell NHL and induces cell apoptosis by antibody-dependent cell-mediated cytotoxicity and complement-dependent cytotoxicity (29,30). Pola binds to CD79b, which is expressed on >95% of B cells in patients with DLBCL. Upon binding and internalization, unconjugated MMAE is released intracellularly, which inhibits cell division and induces apoptosis in proliferating B cells (5). PopPK analyses have shown that some baseline disease-related characteristics (e.g., B cell count and tumor sum of the product diameters) are statistically significant covariates for the PK of R (31), G (32), and pola (9), suggesting the potential for a B cell related drug-drug interaction between pola and R. The pCC approach was used for this analysis with all patients assumed to have R/R disease (the reference covariate value), which allowed unbiased analysis of pooled data across previously untreated and R/R patients for this analysis. Based on the popPK analysis, combination of pola with R/G was associated with mildly higher acMMAE exposures and moderately lower unconjugated MMAE exposures based on the popPK analyses, which could potentially be due to elimination of some CD79b-expressing B-cells by R/G. Thus, target-mediated clearance, although not playing a major role in acMMAE disposition (9), maybe slightly decreased when in combination with R/G, leading to reduced acMMAE degradation and slightly reduced formation of unconjugated MMAE. However, based on the exposure-safety analysis (10), a 24% increase in acMMAE AUC (2350–2910 ng*day/mL) due to R combination was not expected to result in a clinically meaningful increase in the incidence of grade ≥ 2 peripheral neuropathy (13.00–18.09% on average). A decrease in unconjugated MMAE exposures (GMR ~ 0.6; Table IV) is not expected to adversely affect safety or efficacy.

### The Impact of the Manufacturing Process

During the clinical development of pola, materials from two manufacturing process were used to supply clinical trials. The v0.1-derived liquid DP was used for GO29365 (excluding Arm G), DCS4968g, GO27834, and GO29044, whereas v1.0-derived lyophilized DP (the commercial process) was used for Arm G (pola + BR) of study GO29365, and studies GO29833, GO29834, and BO29561. The material comparability exercise was an essential element of pola regulatory filings, given that the body of evidence (PK, efficacy and safety) for approval used the v0.1-derived DP, not the v1.0-derived DP intended for commercialization. Based on the results reported in this paper, there were no clinically meaningful differences in PK between the v1.0-derived lyophilized DP and v0.1-derived liquid DP. These assessments included the cCC method comparing Cycle 6 exposures (AUC, C_max_) between the two DPs, and the population simulation comparing PK time-profiles between the observed and simulated data for v1.0-derived DP. The cCC method corrects for all the known PK covariates to allow unbiased assessment of the impact of the manufacturing process using pooled data across studies/cohorts. Similar to any analysis involving individual post-hoc EBE of the random effects, the results of cCC method could theoretically be affected by parameter shrinkage. To mitigate this risk, population simulations using the population mean and variability estimates (9), not the individual EBE of random effects (thus not subject to parameter shrinkage), were performed to simulate the PK time-profile and to compare to the observed data from the external datasets (assessed by VPC and NPDE). This approach attributed any potential difference in PK between the simulation and observation to the effect of manufacturing process after adjusting for the known covariates based on the model established using v0.1-derived liquid DP data (9). This population simulation approach was also considered to be more stringent and conservative (a “high bar” to meet) for assessing PK similarity between two manufacturing processes, if compared with a potentially alternative, but not undertaken approach, of testing the manufacturing process as covariate in the established popPK model (9) based on the totality of data from two manufacturing processes. In studies GO29833, GO29834 and BO29561, pola + R/G was combined with different drugs (lenalidomide, venetoclax, or atezolizumab, respectively) compared with GO29365 (bendamustine), which could potentially confound the interpretation of the manufacturing process on pola PK. However, the R/R DLBCL patients from Arm G of study GO29365 received pola in combination with the same therapies (BR) as the main randomized cohorts in study GO29365. Therefore, the assessment using Arm G data provided the most relevant comparison of pola PK for v1.0-derived lyophilized *versus* 0.1-derived liquid DP, without the potential confounding effect of different combination drugs. In summary, the popPK approach was successfully utilized to justify material comparability and supported the Biologics License Application filing and global registration instead of conducting a dedicated, material-bridging bioequivalence study in patients which was not feasible as the v0.1 material was no longer available.

Lastly, the non-compartment analysis (NCA) was also used to assess the material comparability (Genentech data on file), and the results are aligned with the conclusion that there are no clinically meaningful differences of pola PK between liquid and lyophilized material. It is worth noting the NCA comparison was limited to a small homogeneous population (Arm G v.s. cohort 1a and Arm C of study GO29365 for patients with R/R DLBCL taking pola 1.8 mg/kg in combination with BR). NCA assessment was not done for pooled data across studies, since this method does not adjust for potential imbalances in PK covariates across studies.

## Conclusions

A two-analyte integrated popPK model for acMMAE and unconjugated MMAE after administration of pola to patients with NHL supports bodyweight-based dosing, and no further pola dose adjustments in subgroups of patients based on major intrinsic and extrinsic factors. After accounting for bodyweight-based dosing, there are no clinically significant differences in the PK of pola in heavier patients (100–146 kg), elderly patients (65–89 years), male or female sex, patients with mild hepatic impairment or mild-to-moderate renal impairment, patients receiving pola in combination with R/G or bendamustine (compared with single-agent pola), and patients receiving v0.1 or v1.0 process derived DP. Limited PK data is available for patients with bodyweight higher than 146 kg, patients with moderate or severe hepatic impairment, or patients with severe renal impairment currently. Ongoing and future clinical trials and real-world data post pola approval may provide dosing guidance for these patients.

### ACKNOWLEDGMENTS AND DISCLOSURES

This study was funded by F. Hoffman-La Roche Ltd. We would like to thank the patients, their families, and the study investigators who participated in the clinical trials used for this analysis, without whom this study would not have been possible. We also acknowledge Xiaobin Li who supported dataset preparations for the analysis presented in this report. Medical writing support was provided by Angela Rogers and Andrea Bothwell of Gardiner-Caldwell Communications, with funding from F. Hoffman-La Roche Ltd.

## Electronic supplementary material

ESM 1 (JPG 1292 kb)

ESM 2 (JPG 3217 kb)

ESM 3 (DOCX 30 kb)

## Abbreviations

ac: Antibody-conjugated
ADC: Antibody–drug conjugate
ALT: Alanine transaminase
AST: Aspartate transaminase
AUC: Area under the concentration-time curve
BR: Bendamustine plus rituximab
BV: Brentuximab vedotin
cCC: Complete covariate correction
CL_INF_: Time-dependent linear clearance
C_max_: Maximum concentration
CrCL: Creatinine clearance
CV%: Coefficient of variation
DLBCL: Diffuse large B cell lymphoma
DP: Drug product
EBE: Empirical Bayes estimates
ECOG: Eastern Cooperative Oncology Group
ESRD: End-stage renal disease
FDA: Food and Drug Administration
FRAC_NS_: Relative fraction of formation
G: Obinutuzumab
GM: Geometric mean
GMR: Geometric mean ratio
IIV: Inter-individual variability
LLOQ: Lower limit of quantification
MC-vc-PAB: Maleimidocaproyl-valine-citrulline-p-aminobenzyloxycarbonyl
MMAE: Monomethyl auristatin E
NCA: Non-compartmental analysis
NCI: National Cancer Institute
NHL: Non-Hodgkin lymphoma
NPDE: Normalized prediction distribution error
pc-VPC: Prediction corrected-visual predictive check
pCC: Partial covariate correction
PK: Pharmacokinetics
pola: Polatuzumab vedotin
popPK: Population PK
Q3W: Every 3 weeks
R: Rituximab
R/R: Relapsed/refractory
RWD: Real-world data
ULN: Upper limit of normal
USPI: United States Prescribing Information
