# Supplementary figures and images for: Application of a Two-Analyte Integrated Population Pharmacokinetic Model to Evaluate the Impact of Intrinsic and Extrinsic Factors on the Pharmacokinetics of Polatuzumab Vedotin in Patients with Non-Hodgkin Lymphoma
Source: Pharm Res. 2020 Dec 1;37(12):252. doi: 10.1007/s11095-020-02933-6 (PMC7708381; doi:10.1007/s11095-020-02933-6)

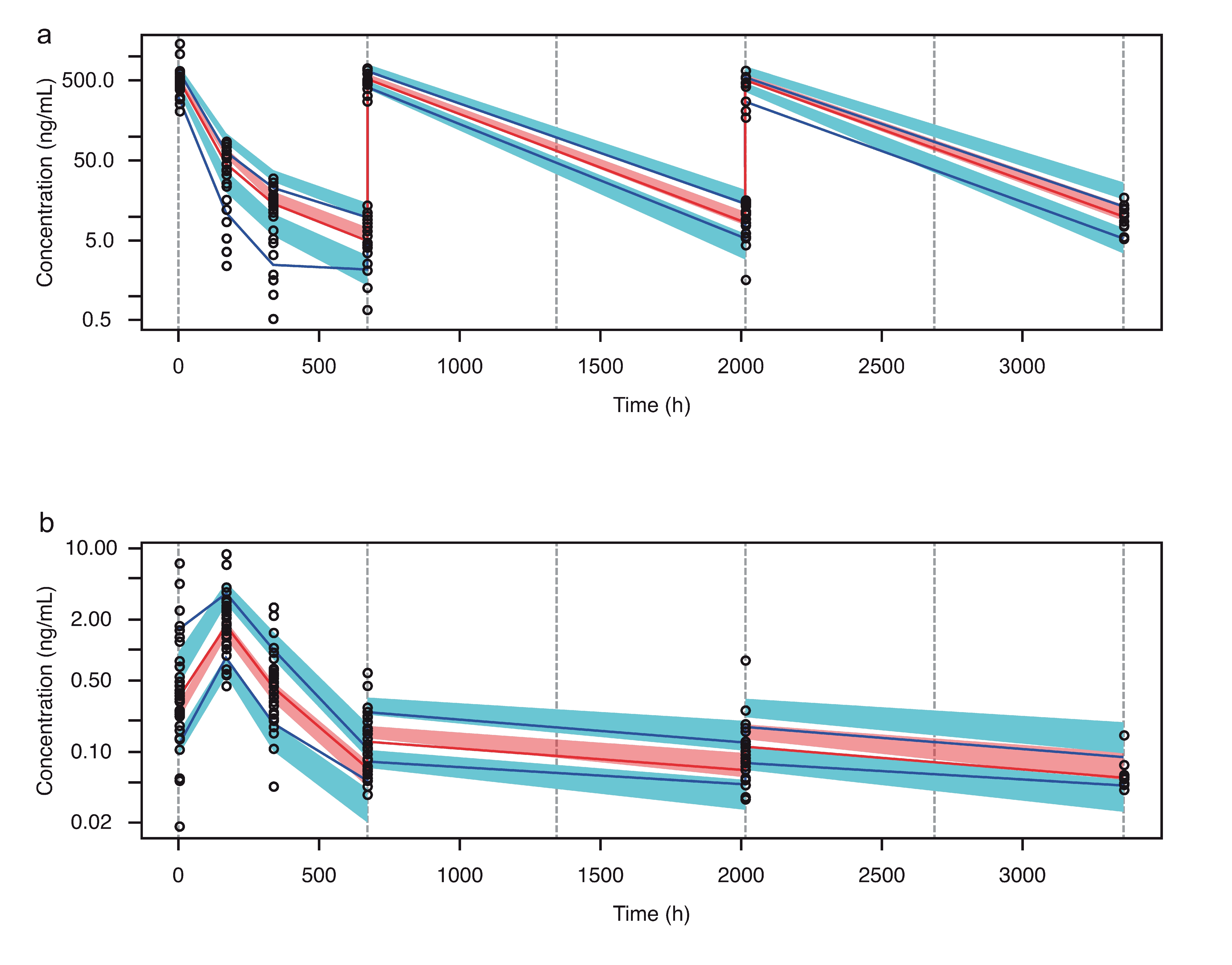

Supplement: Supplementary file 2 — (JPG 3217 kb) [file 11095_2020_2933_MOESM2_ESM.jpg]
