# Supplementary material for: Application of a Two-Analyte Integrated Population Pharmacokinetic Model to Evaluate the Impact of Intrinsic and Extrinsic Factors on the Pharmacokinetics of Polatuzumab Vedotin in Patients with Non-Hodgkin Lymphoma
Source: Pharm Res. 2020 Dec 1;37(12):252. doi: 10.1007/s11095-020-02933-6 (PMC7708381; doi:10.1007/s11095-020-02933-6)
Supplement: Supplementary file 3 — (DOCX 30 kb) [file 11095_2020_2933_MOESM3_ESM.docx]

**SUPPLEMENTARY MATERIAL**

**Application of a two-analyte integrated population PK model to evaluate the impact of intrinsic and extrinsic factors on the pharmacokinetics of polatuzumab vedotin in patients with non-Hodgkin lymphoma**

Dan Lu^1^, Tong Lu^1^, Rong Shi^1^, Leonid Gibiansky^2^, Priya Agarwal^1^, Colby S. Shemesh^1^, Randall C. Dere^1^, Uzor Ogbu^1^, Jamie Hirata^1^, Pascal Chanu^3^, Sandhya Girish^1^, Jin Yan Jin^1^, Chunze Li^1^, Dale Miles^1^

Dan Lu and Tong Lu contributed equally to this work.

**Corresponding author:** Dan Lu

Genentech Research and Early Development, 1 DNA Way, MS46-3a, South San Francisco, CA, 94080, USA

Tel: +1 6504672604

E-mail: lu.dan@gene.com

**Supplementary methods:**

### Prediction-Corrected Visual Predictive Check (pc-VPC) Analysis

Prediction-corrected Visual Predictive Check (pc-VPC) was implemented. Simulations from the model were performed to generate 500 concentration datasets using dosing, sampling and covariates of the original data. The original concentrations and the simulated values were prediction-corrected as follows:

pcY_ij_ = Y_ij_/PRED_ij_*PRED_bin_ ,

where Y_ij_ is the observation or prediction for the i^th^ individual and j^th^ time point, pcY_ij_ is the prediction-corrected observation or prediction, PRED_ij_ is the typical population prediction for the i^th^ individual and j^th^ time point, and PRED_bin_ is median of typical population predictions of the independent variable for the specific nominal time point.

For each dataset (original and simulated) and each bin, 10^th^, 50^th^, and 90^th^ percentiles of prediction-corrected concentration values were calculated. For each of the calculated percentiles of the simulated values the 80% confidence intervals were constructed by calculating 10^th^ and 90^th^ percentiles across 500 simulations.

The observed percentiles and confidence intervals for the simulated percentiles were plotted versus time together with the prediction-corrected observed values.

### Normalized Prediction Distribution Errors (NPDE) Analysis

The normalized prediction distribution errors (NPDE) procedure was implemented. For each observation, 500 NPDE values were derived. The prediction discrepancy was defined as the percentile of the observation in the predictive distribution simulated from the model. Prediction discrepancies are correlated within an individual. Therefore, mean and variance of the predicted observations estimated empirically from simulations were used to obtain an uncorrelated NPDE metric (in NONMEM). NPDE values should be normally distributed. If the PK of the v1.0-derived DP was consistent with the model prediction, it would be expected that approximately 50% of the NPDE values would be above the 50^th^ percentile of a standard normal distribution of *N* (0,1), 10% of the NPDE values are above the 90^th^ percentile, and 10% of the NPDE values are below the 10^th^ percentile. Plots of NPDE versus covariates should not exhibit any consistent trends. NPDE values were plotted versus time from the first dose, time after dose, population predictions, and important covariates, overall and where appropriate by subsets of the data. Percentages of points below 10^th^ percentile and above 50^th^ and 90^th^ percentiles of a standard normal distribution were reported.

**Supplementary Table I** NCI criteria for hepatic function impairment and number of patients in each category from polatuzumab vedotin clinical studies (DCS4968g, GO27834, GO29365 and GO29044) [15]

| **Hepatic function (group number)** | **Bilirubin** | **AST** | **N** | **Range of bilirubin, mg/dL** | **Range of AST, IU/L** | **Range of ALT, IU/L** |
| --- | --- | --- | --- | --- | --- | --- |
| Normal (A) | ≤ULN | ≤ULN | 399 | 0.142–1.000 | 0.236–1.00 | 0.104–2.650 |
| Mild impairment (B1) | ≤ULN | >ULN | 45 | 0.154–1.000 | 1.000–5.82^a^ | 0.167–5.650^a^ |
| Mild impairment (B2) | >ULN and ≤1.5 ULN | any | 9 | 1.040–1.460 | 0.400–2.510 | 0.204–1.230 |
| Moderate impairment (C) | >1.5 ULN and ≤3.0 ULN | any | 5^b^ | 1.600–2.100 | 0.422–2.140 | 0.444–1.620 |
| Severe impairment (D) | >3 ULN | any | 0 | NA | NA | NA |
| Liver transplant (E) | any | any | 0 | NA | NA | NA |

^a^One patient had a ratio of AST to ULN of 5.82, and another patient had a ratio of ALT to ULN of 5.65 and AST to ULN of 4.86. When both patients were removed, the range of AST ratio to ULN was [1.000–2.220 ULN], and the range of ALT ratio to ULN was [0.167–2.460 ULN], which are overall aligned with the study exclusion criteria of >2.5 ULN for AST and ALT.

^b^Of the five patients, three had total bilirubin elevation due to Gilbert’s disease, thus only two patients were considered to have moderate hepatic impairment.

Abbreviations: ALT, alanine transaminase; AST, aspartate transaminase; NA, not applicable; NCI, National Cancer Institute; ULN, upper limit of the normal range.

**Supplementary Table II** Distribution of patients with renal impairment in studies DCS4968g, GO27834, GO29044 and GO29365 (pooled data based on the population PK dataset)

|  | **Renal impairment status (specified CrCL range, mL/min)** | | | | |
| --- | --- | --- | --- | --- | --- |
|  | **Normal (≥90)** | **Mild**  **(60–89)** | **Moderate (30–59)** | **Severe (15–29)** | **ESRD (<15)** |
| Number of patients^a^ | 185 | 161 | 109 | 3 | 0 |
| Median CrCL (range), mL/min | 107 (90.3–270) | 75.7 (60.4–90) | 52.3 (30.1–60) | 28.7 (28.4–29.5) | – |

^a^Renal impairment status for 2/460 patients was missing.

Abbreviations: CrCL, creatinine clearance; ESRD, end-stage renal disease.

**Supplementary Table III** NPDE assessment with patients dosed with v1.0-derived lyophilized polatuzumab vedotin. The results showed the percentage of values above or below certain percentiles of a standard normal distribution N (0,1)

|  | **Analyte** | **Above 50^th^ percentile (%)** | **Above 90^th^ percentile (%)** | **Below 10^th^ percentile (%)** |
| --- | --- | --- | --- | --- |
| *N* = 106 (GO29833, GO29834 and GO29561 studies) | acMMAE | 45 | 5 | 13 |
|  | Unconjugated MMAE | 46 | 11 | 13 |
| *N* = 42 (Arm G of the GO29365 study) | acMMAE | 50 | 6 | 12 |
|  | Unconjugated MMAE | 37 | 9 | 15 |

If observed pharmacokinetic values were consistent with the model, approximately 50% and 10% of NPDE values were expected to be above the 50^th^ and 90^th^ percentiles, respectively, and 10% were expected to be below the 10^th^ percentile of a standard normal distribution of N (0,1).

Abbreviation: acMMAE, antibody-conjugated monomethyl auristatin E; MMAE, monomethyl auristatin E; NPDE, normalized prediction distribution error.

**Supplementary Fig. 1** Goodness-of-Fit diagnosis for studies GO29833, GO29834, and BO29561 used as external dataset for assessing impact of material on PK by population PK analysis **a**) acMMAE, and **b**) unconjugated MMAE.

Abbreviations: CWRES, conditional weighted residuals; DV, observations; IPRED, individual predictions; PRED, population predictions; TAD, time after dose.

**Supplementary Fig. 2** Goodness-of-Fit diagnosis for Arm G of the GO29365 study used as external dataset for assessing impact of material on PK by population PK analysis **a**) acMMAE, and **b**) unconjugated MMAE.

Abbreviations: CWRES, conditional weighted residuals; DV, observations; IPRED, individual predictions; PRED, population predictions; TAD, time after dose.

**Supplementary Fig. 3** Prediction-corrected visual predictive check (pc-VPC) plots of **a**) acMMAE from combined GO29833 and BO29561 data, **b**) unconjugated MMAE from combined GO29833 and BO29561 data, **c**) acMMAE from Arm G of the GO29365 study, and **d**) unconjugated MMAE from Arm G of the GO29365 study, with all patients dosed with v1.0-derived lyophilized polatuzumab vedotin administered with Q3W dosing.

Note: Points are prediction-corrected concentrations plotted versus time after the first polatuzumab vedotin dose. The lines show median (red), and the 10^th^ and 90^th^ percentiles (blue) of the prediction-corrected concentrations. The shaded regions show the 80% CI on these percentiles obtained by simulations of 500 trials with dosing, PK sampling, and the baseline covariate values of the dataset of pooled PK data from the GO29833 and BO29561 studies. Dashed perpendicular line: polatuzumab vedotin dosing time.

Abbreviations: ac, antibody-conjugated; CI, confidence interval; MMAE, monomethyl auristatin E; pc, prediction corrected; PK, pharmacokinetic; Q3W, every 3 weeks.

**Supplementary Fig. 4** Prediction-corrected visual predictive check plots for **a**) acMMAE, and **b**) unconjugated MMAE from patients in the GO29834 study after v1.0-derived lyophilized polatuzumab vedotin administered with Q4W dosing.

Note: Points are prediction-corrected concentrations plotted versus time after the first polatuzumab vedotin dose. The lines show median (red), and the 10^th^ and 90^th^ percentiles (blue) of the prediction-corrected concentrations. The shaded regions show the 80% CI on these percentiles obtained by simulations of 500 trials with dosing, PK sampling, and the baseline covariate values of the dataset of pooled PK data from the GO29834 study. Dashed perpendicular line: polatuzumab vedotin dosing time.

Abbreviations: ac, antibody-conjugated; CI, confidence interval; MMAE, monomethyl auristatin E; pc, prediction corrected; PK, pharmacokinetic; Q4W, every 4 weeks.
